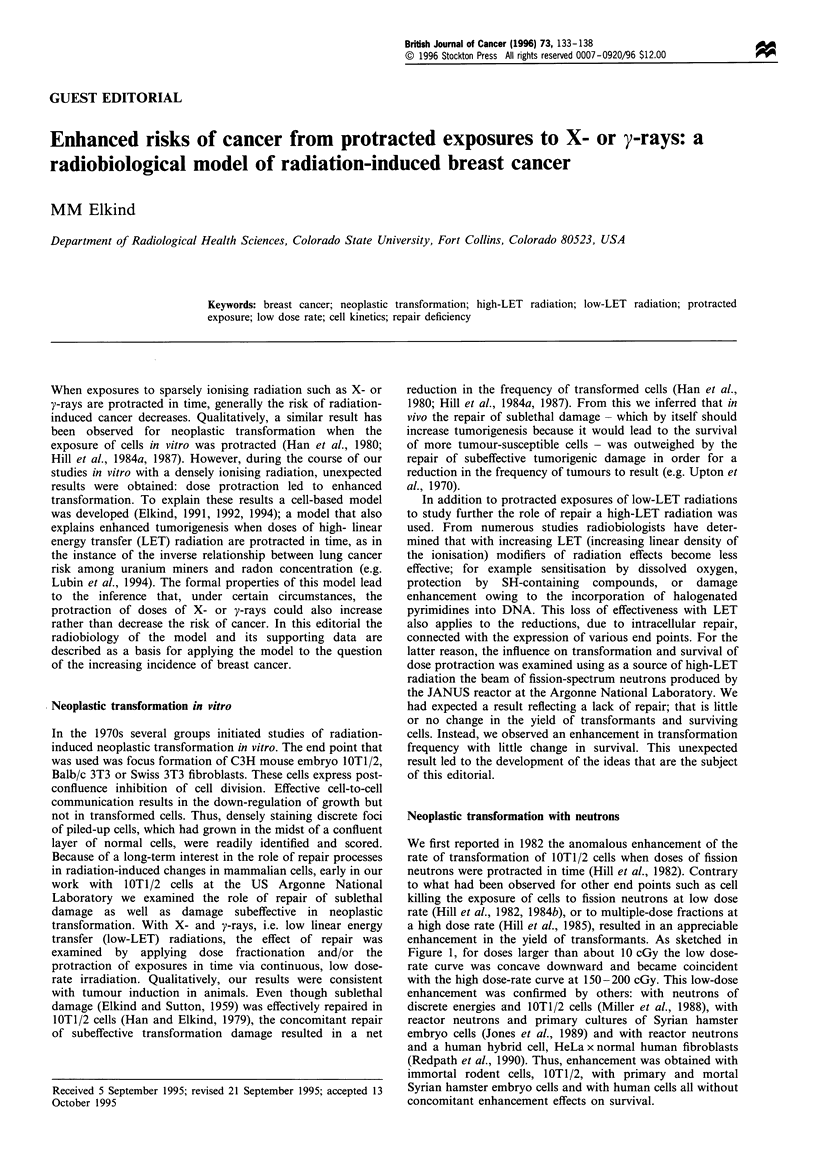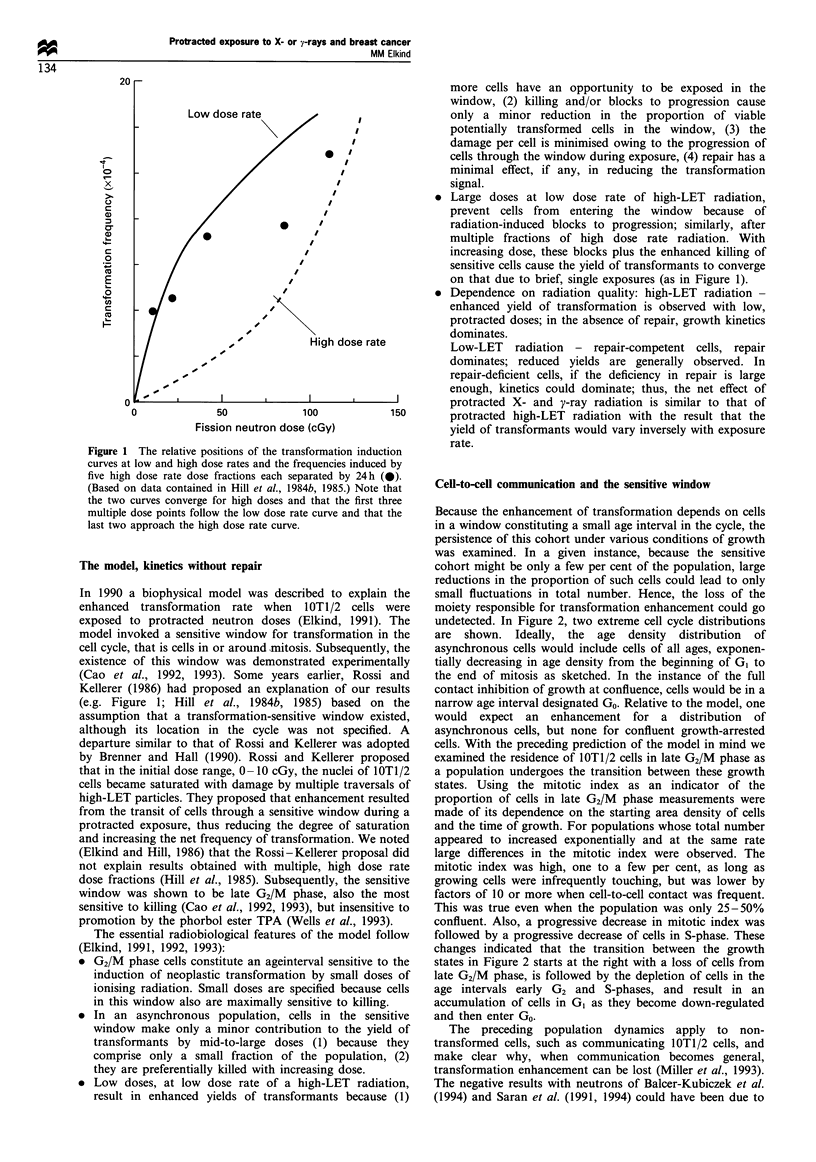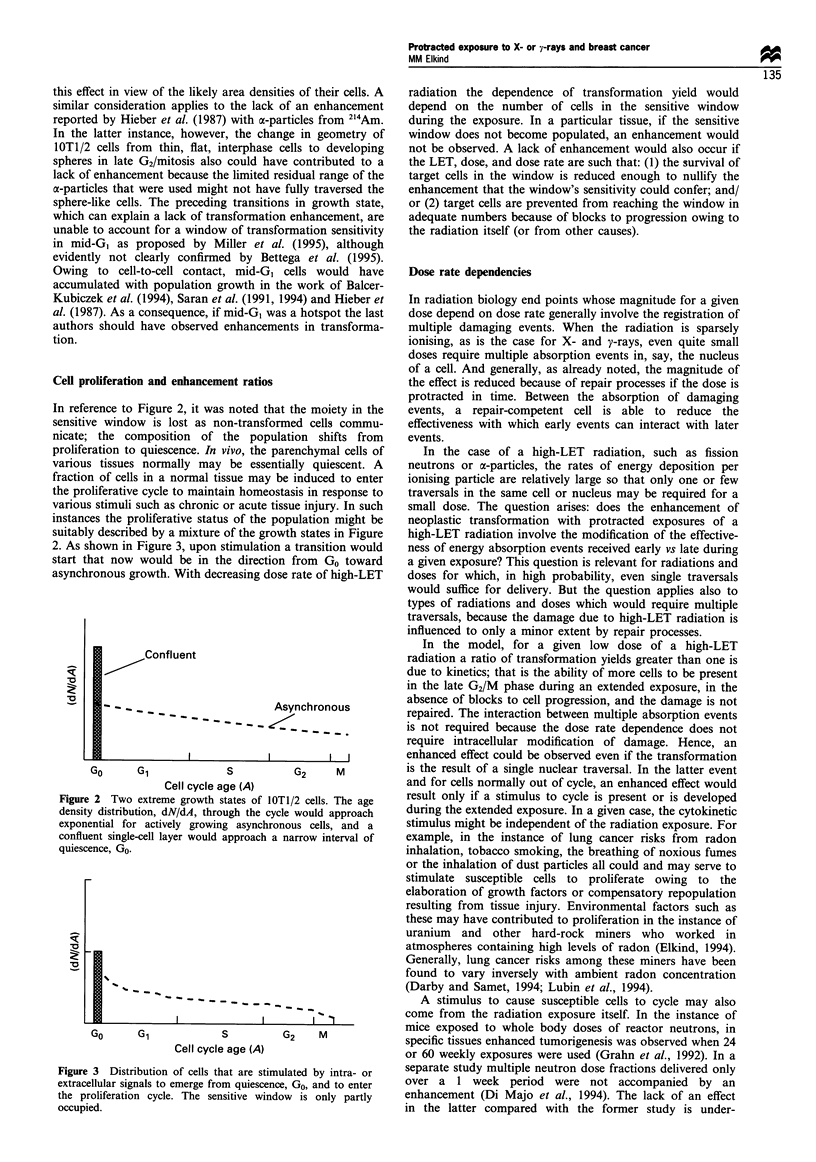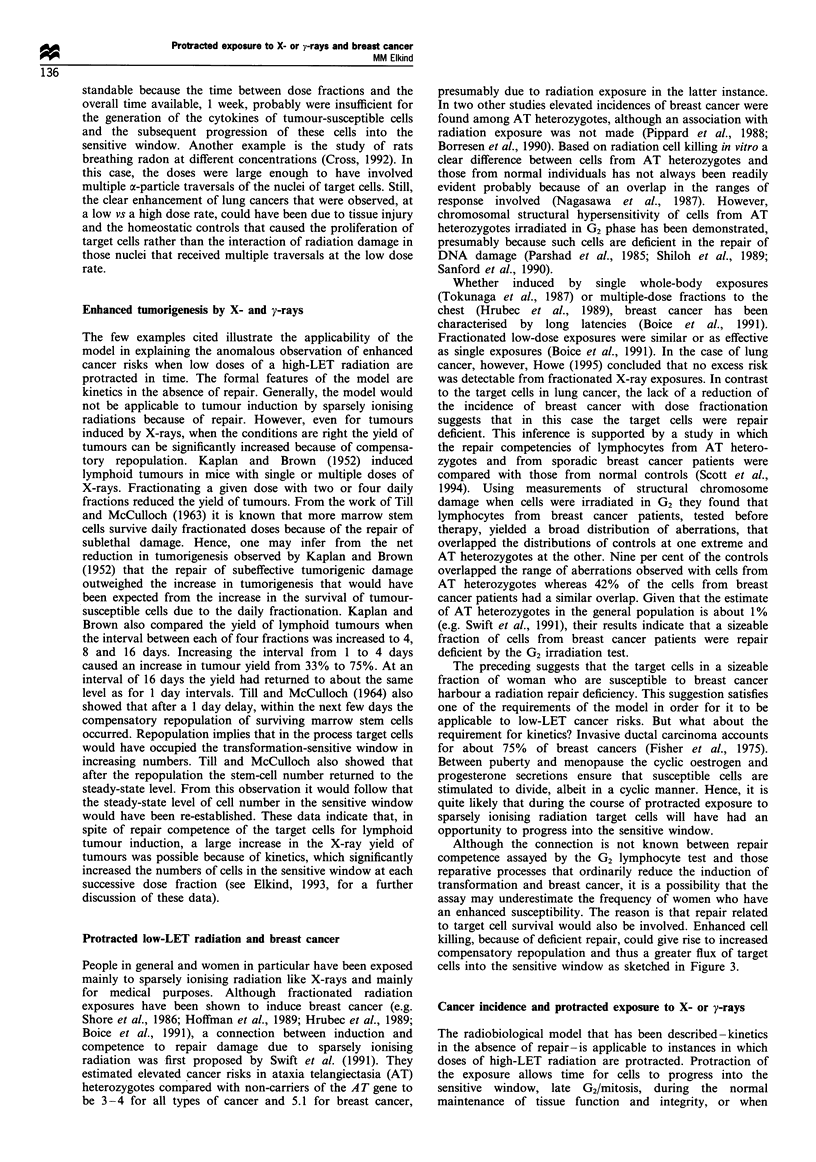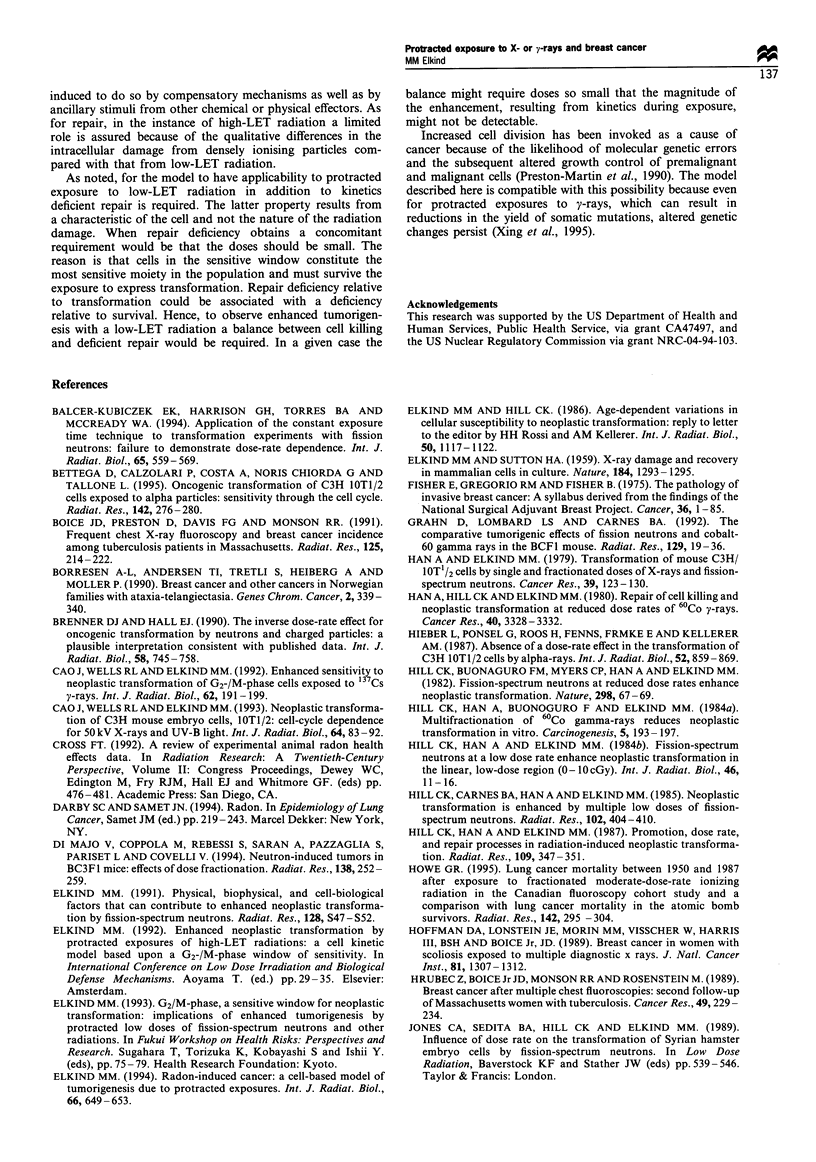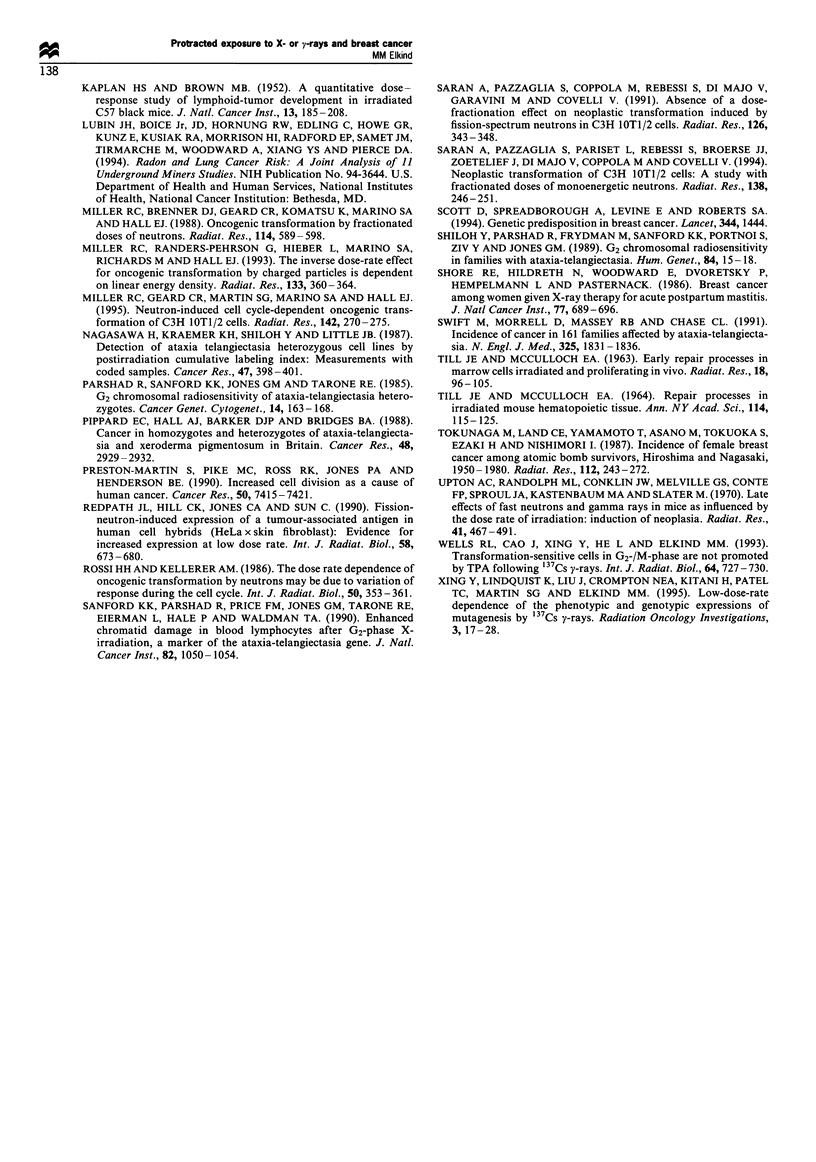# Enhanced risks of cancer from protracted exposures to X- or gamma-rays: a radiobiological model of radiation-induced breast cancer.

**DOI:** 10.1038/bjc.1996.25

**Published:** 1996-01

**Authors:** M. M. Elkind

**Affiliations:** Department of Radiological Health Sciences, Colorado State University, Fort Collins 80523, USA.


British Journal of Cancer (1996) 73, 133–138
© 1996 Stockton Press All rights reserved 0007-0920/96 $12.00

GUEST EDITORIAL

### Enhanced risks of cancer from protracted exposures to X- or $\gamma$-rays: a radiobiological model of radiation-induced breast cancer

MM Elkind

*Department of Radiological Health Sciences, Colorado State University, Fort Collins, Colorado 80523, USA*

**Keywords:** breast cancer; neoplastic transformation; high-LET radiation; low-LET radiation; protracted exposure; low dose rate; cell kinetics; repair deficiency

When exposures to sparsely ionising radiation such as X- or $\gamma$-rays are protracted in time, generally the risk of radiation-induced cancer decreases. Qualitatively, a similar result has been observed for neoplastic transformation when the exposure of cells *in vitro* was protracted (Han *et al.*, 1980; Hill *et al.*, 1984*a*, 1987). However, during the course of our studies *in vitro* with a densely ionising radiation, unexpected results were obtained: dose protraction led to enhanced transformation. To explain these results a cell-based model was developed (Elkind, 1991, 1992, 1994); a model that also explains enhanced tumorigenesis when doses of high- linear energy transfer (LET) radiation are protracted in time, as in the instance of the inverse relationship between lung cancer risk among uranium miners and radon concentration (e.g. Lubin *et al.*, 1994). The formal properties of this model lead to the inference that, under certain circumstances, the protraction of doses of X- or $\gamma$-rays could also increase rather than decrease the risk of cancer. In this editorial the radiobiology of the model and its supporting data are described as a basis for applying the model to the question of the increasing incidence of breast cancer.

### Neoplastic transformation *in vitro*

In the 1970s several groups initiated studies of radiation-induced neoplastic transformation *in vitro*. The end point that was used was focus formation of C3H mouse embryo 10T1/2, Balb/c 3T3 or Swiss 3T3 fibroblasts. These cells express post-confluence inhibition of cell division. Effective cell-to-cell communication results in the down-regulation of growth but not in transformed cells. Thus, densely staining discrete foci of piled-up cells, which had grown in the midst of a confluent layer of normal cells, were readily identified and scored. Because of a long-term interest in the role of repair processes in radiation-induced changes in mammalian cells, early in our work with 10T1/2 cells at the US Argonne National Laboratory we examined the role of repair of sublethal damage as well as damage subeffective in neoplastic transformation. With X- and $\gamma$-rays, i.e. low linear energy transfer (low-LET) radiations, the effect of repair was examined by applying dose fractionation and/or the protraction of exposures in time via continuous, low dose-rate irradiation. Qualitatively, our results were consistent with tumour induction in animals. Even though sublethal damage (Elkind and Sutton, 1959) was effectively repaired in 10T1/2 cells (Han and Elkind, 1979), the concomitant repair of subeffective transformation damage resulted in a net reduction in the frequency of transformed cells (Han *et al.*, 1980; Hill *et al.*, 1984*a*, 1987). From this we inferred that *in vivo* the repair of sublethal damage – which by itself should increase tumorigenesis because it would lead to the survival of more tumour-susceptible cells – was outweighed by the repair of subeffective tumorigenic damage in order for a reduction in the frequency of tumours to result (e.g. Upton *et al.*, 1970).

In addition to protracted exposures of low-LET radiations to study further the role of repair a high-LET radiation was used. From numerous studies radiobiologists have determined that with increasing LET (increasing linear density of the ionisation) modifiers of radiation effects become less effective; for example sensitisation by dissolved oxygen, protection by SH-containing compounds, or damage enhancement owing to the incorporation of halogenated pyrimidines into DNA. This loss of effectiveness with LET also applies to the reductions, due to intracellular repair, connected with the expression of various end points. For the latter reason, the influence on transformation and survival of dose protraction was examined using as a source of high-LET radiation the beam of fission-spectrum neutrons produced by the JANUS reactor at the Argonne National Laboratory. We had expected a result reflecting a lack of repair; that is little or no change in the yield of transformants and surviving cells. Instead, we observed an enhancement in transformation frequency with little change in survival. This unexpected result led to the development of the ideas that are the subject of this editorial.

### Neoplastic transformation with neutrons

We first reported in 1982 the anomalous enhancement of the rate of transformation of 10T1/2 cells when doses of fission neutrons were protracted in time (Hill *et al.*, 1982). Contrary to what had been observed for other end points such as cell killing the exposure of cells to fission neutrons at low dose rate (Hill *et al.*, 1982, 1984*b*), or to multiple-dose fractions at a high dose rate (Hill *et al.*, 1985), resulted in an appreciable enhancement in the yield of transformants. As sketched in Figure 1, for doses larger than about 10 cGy the low dose-rate curve was concave downward and became coincident with the high dose-rate curve at 150–200 cGy. This low-dose enhancement was confirmed by others: with neutrons of discrete energies and 10T1/2 cells (Miller *et al.*, 1988), with reactor neutrons and primary cultures of Syrian hamster embryo cells (Jones *et al.*, 1989) and with reactor neutrons and a human hybrid cell, HeLa × normal human fibroblasts (Redpath *et al.*, 1990). Thus, enhancement was obtained with immortal rodent cells, 10T1/2, with primary and mortal Syrian hamster embryo cells and with human cells all without concomitant enhancement effects on survival.

Received 5 September 1995; revised 21 September 1995; accepted 13 October 1995

**Figure 1** The relative positions of the transformation induction curves at low and high dose rates and the frequencies induced by five high dose rate dose fractions each separated by 24 h (●). (Based on data contained in Hill *et al.*, 1984*b*, 1985.) Note that the two curves converge for high doses and that the first three multiple dose points follow the low dose rate curve and that the last two approach the high dose rate curve.

### The model, kinetics without repair

In 1990 a biophysical model was described to explain the enhanced transformation rate when 10T1/2 cells were exposed to protracted neutron doses (Elkind, 1991). The model invoked a sensitive window for transformation in the cell cycle, that is cells in or around mitosis. Subsequently, the existence of this window was demonstrated experimentally (Cao *et al.*, 1992, 1993). Some years earlier, Rossi and Kellerer (1986) had proposed an explanation of our results (e.g. Figure 1; Hill *et al.*, 1984*b*, 1985) based on the assumption that a transformation-sensitive window existed, although its location in the cycle was not specified. A departure similar to that of Rossi and Kellerer was adopted by Brenner and Hall (1990). Rossi and Kellerer proposed that in the initial dose range, 0–10 cGy, the nuclei of 10T1/2 cells became saturated with damage by multiple traversals of high-LET particles. They proposed that enhancement resulted from the transit of cells through a sensitive window during a protracted exposure, thus reducing the degree of saturation and increasing the net frequency of transformation. We noted (Elkind and Hill, 1986) that the Rossi–Kellerer proposal did not explain results obtained with multiple, high dose rate dose fractions (Hill *et al.*, 1985). Subsequently, the sensitive window was shown to be late $G_2$/M phase, also the most sensitive to killing (Cao *et al.*, 1992, 1993), but insensitive to promotion by the phorbol ester TPA (Wells *et al.*, 1993).

The essential radiobiological features of the model follow (Elkind, 1991, 1992, 1993):

- $G_2$/M phase cells constitute an ageinterval sensitive to the induction of neoplastic transformation by small doses of ionising radiation. Small doses are specified because cells in this window also are maximally sensitive to killing.
- In an asynchronous population, cells in the sensitive window make only a minor contribution to the yield of transformants by mid-to-large doses (1) because they comprise only a small fraction of the population, (2) they are preferentially killed with increasing dose.
- Low doses, at low dose rate of a high-LET radiation, result in enhanced yields of transformants because (1) more cells have an opportunity to be exposed in the window, (2) killing and/or blocks to progression cause only a minor reduction in the proportion of viable potentially transformed cells in the window, (3) the damage per cell is minimised owing to the progression of cells through the window during exposure, (4) repair has a minimal effect, if any, in reducing the transformation signal.
- Large doses at low dose rate of high-LET radiation, prevent cells from entering the window because of radiation-induced blocks to progression; similarly, after multiple fractions of high dose rate radiation. With increasing dose, these blocks plus the enhanced killing of sensitive cells cause the yield of transformants to converge on that due to brief, single exposures (as in Figure 1).
- Dependence on radiation quality: high-LET radiation – enhanced yield of transformation is observed with low, protracted doses; in the absence of repair, growth kinetics dominates.

  Low-LET radiation – repair-competent cells, repair dominates; reduced yields are generally observed. In repair-deficient cells, if the deficiency in repair is large enough, kinetics could dominate; thus, the net effect of protracted X- and γ-ray radiation is similar to that of protracted high-LET radiation with the result that the yield of transformants would vary inversely with exposure rate.

### Cell-to-cell communication and the sensitive window

Because the enhancement of transformation depends on cells in a window constituting a small age interval in the cycle, the persistence of this cohort under various conditions of growth was examined. In a given instance, because the sensitive cohort might be only a few per cent of the population, large reductions in the proportion of such cells could lead to only small fluctuations in total number. Hence, the loss of the moiety responsible for transformation enhancement could go undetected. In Figure 2, two extreme cell cycle distributions are shown. Ideally, the age density distribution of asynchronous cells would include cells of all ages, exponentially decreasing in age density from the beginning of $G_1$ to the end of mitosis as sketched. In the instance of the full contact inhibition of growth at confluence, cells would be in a narrow age interval designated $G_0$. Relative to the model, one would expect an enhancement for a distribution of asynchronous cells, but none for confluent growth-arrested cells. With the preceding prediction of the model in mind we examined the residence of 10T1/2 cells in late $G_2$/M phase as a population undergoes the transition between these growth states. Using the mitotic index as an indicator of the proportion of cells in late $G_2$/M phase measurements were made of its dependence on the starting area density of cells and the time of growth. For populations whose total number appeared to increased exponentially and at the same rate large differences in the mitotic index were observed. The mitotic index was high, one to a few per cent, as long as growing cells were infrequently touching, but was lower by factors of 10 or more when cell-to-cell contact was frequent. This was true even when the population was only 25–50% confluent. Also, a progressive decrease in mitotic index was followed by a progressive decrease of cells in S-phase. These changes indicated that the transition between the growth states in Figure 2 starts at the right with a loss of cells from late $G_2$/M phase, is followed by the depletion of cells in the age intervals early $G_2$ and S-phases, and result in an accumulation of cells in $G_1$ as they become down-regulated and then enter $G_0$.

The preceding population dynamics apply to non-transformed cells, such as communicating 10T1/2 cells, and make clear why, when communication becomes general, transformation enhancement can be lost (Miller *et al.*, 1993). The negative results with neutrons of Balcer-Kubiczek *et al.* (1994) and Saran *et al.* (1991, 1994) could have been due to this effect in view of the likely area densities of their cells. A similar consideration applies to the lack of an enhancement reported by Hieber *et al.* (1987) with α-particles from $^{214}$Am. In the latter instance, however, the change in geometry of 10T1/2 cells from thin, flat, interphase cells to developing spheres in late $G_2$/mitosis also could have contributed to a lack of enhancement because the limited residual range of the α-particles that were used might not have fully traversed the sphere-like cells. The preceding transitions in growth state, which can explain a lack of transformation enhancement, are unable to account for a window of transformation sensitivity in mid-$G_1$ as proposed by Miller *et al.* (1995), although evidently not clearly confirmed by Bettega *et al.* (1995). Owing to cell-to-cell contact, mid-$G_1$ cells would have accumulated with population growth in the work of Balcer-Kubiczek *et al.* (1994), Saran *et al.* (1991, 1994) and Hieber *et al.* (1987). As a consequence, if mid-$G_1$ was a hotspot the last authors should have observed enhancements in transformation.

### Cell proliferation and enhancement ratios

In reference to Figure 2, it was noted that the moiety in the sensitive window is lost as non-transformed cells communicate; the composition of the population shifts from proliferation to quiescence. *In vivo*, the parenchymal cells of various tissues normally may be essentially quiescent. A fraction of cells in a normal tissue may be induced to enter the proliferative cycle to maintain homeostasis in response to various stimuli such as chronic or acute tissue injury. In such instances the proliferative status of the population might be suitably described by a mixture of the growth states in Figure 2. As shown in Figure 3, upon stimulation a transition would start that now would be in the direction from $G_0$ toward asynchronous growth. With decreasing dose rate of high-LET radiation the dependence of transformation yield would depend on the number of cells in the sensitive window during the exposure. In a particular tissue, if the sensitive window does not become populated, an enhancement would not be observed. A lack of enhancement would also occur if the LET, dose, and dose rate are such that: (1) the survival of target cells in the window is reduced enough to nullify the enhancement that the window's sensitivity could confer; and/or (2) target cells are prevented from reaching the window in adequate numbers because of blocks to progression owing to the radiation itself (or from other causes).

**Figure 2** Two extreme growth states of 10T1/2 cells. The age density distribution, d$N$/d$A$, through the cycle would approach exponential for actively growing asynchronous cells, and a confluent single-cell layer would approach a narrow interval of quiescence, $G_0$.

**Figure 3** Distribution of cells that are stimulated by intra- or extracellular signals to emerge from quiescence, $G_0$, and to enter the proliferation cycle. The sensitive window is only partly occupied.

### Dose rate dependencies

In radiation biology end points whose magnitude for a given dose depend on dose rate generally involve the registration of multiple damaging events. When the radiation is sparsely ionising, as is the case for X- and γ-rays, even quite small doses require multiple absorption events in, say, the nucleus of a cell. And generally, as already noted, the magnitude of the effect is reduced because of repair processes if the dose is protracted in time. Between the absorption of damaging events, a repair-competent cell is able to reduce the effectiveness with which early events can interact with later events.

In the case of a high-LET radiation, such as fission neutrons or α-particles, the rates of energy deposition per ionising particle are relatively large so that only one or few traversals in the same cell or nucleus may be required for a small dose. The question arises: does the enhancement of neoplastic transformation with protracted exposures of a high-LET radiation involve the modification of the effectiveness of energy absorption events received early *vs* late during a given exposure? This question is relevant for radiations and doses for which, in high probability, even single traversals would suffice for delivery. But the question applies also to types of radiations and doses which would require multiple traversals, because the damage due to high-LET radiation is influenced to only a minor extent by repair processes.

In the model, for a given low dose of a high-LET radiation a ratio of transformation yields greater than one is due to kinetics; that is the ability of more cells to be present in the late $G_2$/M phase during an extended exposure, in the absence of blocks to cell progression, and the damage is not repaired. The interaction between multiple absorption events is not required because the dose rate dependence does not require intracellular modification of damage. Hence, an enhanced effect could be observed even if the transformation is the result of a single nuclear traversal. In the latter event and for cells normally out of cycle, an enhanced effect would result only if a stimulus to cycle is present or is developed during the extended exposure. In a given case, the cytokinetic stimulus might be independent of the radiation exposure. For example, in the instance of lung cancer risks from radon inhalation, tobacco smoking, the breathing of noxious fumes or the inhalation of dust particles all could and may serve to stimulate susceptible cells to proliferate owing to the elaboration of growth factors or compensatory repopulation resulting from tissue injury. Environmental factors such as these may have contributed to proliferation in the instance of uranium and other hard-rock miners who worked in atmospheres containing high levels of radon (Elkind, 1994). Generally, lung cancer risks among these miners have been found to vary inversely with ambient radon concentration (Darby and Samet, 1994; Lubin *et al.*, 1994).

A stimulus to cause susceptible cells to cycle may also come from the radiation exposure itself. In the instance of mice exposed to whole body doses of reactor neutrons, in specific tissues enhanced tumorigenesis was observed when 24 or 60 weekly exposures were used (Grahn *et al.*, 1992). In a separate study multiple neutron dose fractions delivered only over a 1 week period were not accompanied by an enhancement (Di Majo *et al.*, 1994). The lack of an effect in the latter compared with the former study is understandable because the time between dose fractions and the overall time available, 1 week, probably were insufficient for the generation of the cytokines of tumour-susceptible cells and the subsequent progression of these cells into the sensitive window. Another example is the study of rats breathing radon at different concentrations (Cross, 1992). In this case, the doses were large enough to have involved multiple α-particle traversals of the nuclei of target cells. Still, the clear enhancement of lung cancers that were observed, at a low *vs* a high dose rate, could have been due to tissue injury and the homeostatic controls that caused the proliferation of target cells rather than the interaction of radiation damage in those nuclei that received multiple traversals at the low dose rate.

### Enhanced tumorigenesis by X- and γ-rays

The few examples cited illustrate the applicability of the model in explaining the anomalous observation of enhanced cancer risks when low doses of a high-LET radiation are protracted in time. The formal features of the model are kinetics in the absence of repair. Generally, the model would not be applicable to tumour induction by sparsely ionising radiations because of repair. However, even for tumours induced by X-rays, when the conditions are right the yield of tumours can be significantly increased because of compensatory repopulation. Kaplan and Brown (1952) induced lymphoid tumours in mice with single or multiple doses of X-rays. Fractionating a given dose with two or four daily fractions reduced the yield of tumours. From the work of Till and McCulloch (1963) it is known that more marrow stem cells survive daily fractionated doses because of the repair of sublethal damage. Hence, one may infer from the net reduction in tumorigenesis observed by Kaplan and Brown (1952) that the repair of subeffective tumorigenic damage outweighed the increase in tumorigenesis that would have been expected from the increase in the survival of tumour-susceptible cells due to the daily fractionation. Kaplan and Brown also compared the yield of lymphoid tumours when the interval between each of four fractions was increased to 4, 8 and 16 days. Increasing the interval from 1 to 4 days caused an increase in tumour yield from 33% to 75%. At an interval of 16 days the yield had returned to about the same level as for 1 day intervals. Till and McCulloch (1964) also showed that after a 1 day delay, within the next few days the compensatory repopulation of surviving marrow stem cells occurred. Repopulation implies that in the process target cells would have occupied the transformation-sensitive window in increasing numbers. Till and McCulloch also showed that after the repopulation the stem-cell number returned to the steady-state level. From this observation it would follow that the steady-state level of cell number in the sensitive window would have been re-established. These data indicate that, in spite of repair competence of the target cells for lymphoid tumour induction, a large increase in the X-ray yield of tumours was possible because of kinetics, which significantly increased the numbers of cells in the sensitive window at each successive dose fraction (see Elkind, 1993, for a further discussion of these data).

### Protracted low-LET radiation and breast cancer

People in general and women in particular have been exposed mainly to sparsely ionising radiation like X-rays and mainly for medical purposes. Although fractionated radiation exposures have been shown to induce breast cancer (e.g. Shore *et al.*, 1986; Hoffman *et al.*, 1989; Hrubec *et al.*, 1989; Boice *et al.*, 1991), a connection between induction and competence to repair damage due to sparsely ionising radiation was first proposed by Swift *et al.* (1991). They estimated elevated cancer risks in ataxia telangiectasia (AT) heterozygotes compared with non-carriers of the *AT* gene to be 3–4 for all types of cancer and 5.1 for breast cancer, presumably due to radiation exposure in the latter instance. In two other studies elevated incidences of breast cancer were found among AT heterozygotes, although an association with radiation exposure was not made (Pippard *et al.*, 1988; Borresen *et al.*, 1990). Based on radiation cell killing *in vitro* a clear difference between cells from AT heterozygotes and those from normal individuals has not always been readily evident probably because of an overlap in the ranges of response involved (Nagasawa *et al.*, 1987). However, chromosomal structural hypersensitivity of cells from AT heterozygotes irradiated in $G_2$ phase has been demonstrated, presumably because such cells are deficient in the repair of DNA damage (Parshad *et al.*, 1985; Shiloh *et al.*, 1989; Sanford *et al.*, 1990).

Whether induced by single whole-body exposures (Tokunaga *et al.*, 1987) or multiple-dose fractions to the chest (Hrubec *et al.*, 1989), breast cancer has been characterised by long latencies (Boice *et al.*, 1991). Fractionated low-dose exposures were similar or as effective as single exposures (Boice *et al.*, 1991). In the case of lung cancer, however, Howe (1995) concluded that no excess risk was detectable from fractionated X-ray exposures. In contrast to the target cells in lung cancer, the lack of a reduction of the incidence of breast cancer with dose fractionation suggests that in this case the target cells were repair deficient. This inference is supported by a study in which the repair competencies of lymphocytes from AT heterozygotes and from sporadic breast cancer patients were compared with those from normal controls (Scott *et al.*, 1994). Using measurements of structural chromosome damage when cells were irradiated in $G_2$ they found that lymphocytes from breast cancer patients, tested before therapy, yielded a broad distribution of aberrations, that overlapped the distributions of controls at one extreme and AT heterozygotes at the other. Nine per cent of the controls overlapped the range of aberrations observed with cells from AT heterozygotes whereas 42% of the cells from breast cancer patients had a similar overlap. Given that the estimate of AT heterozygotes in the general population is about 1% (e.g. Swift *et al.*, 1991), their results indicate that a sizeable fraction of cells from breast cancer patients were repair deficient by the $G_2$ irradiation test.

The preceding suggests that the target cells in a sizeable fraction of woman who are susceptible to breast cancer harbour a radiation repair deficiency. This suggestion satisfies one of the requirements of the model in order for it to be applicable to low-LET cancer risks. But what about the requirement for kinetics? Invasive ductal carcinoma accounts for about 75% of breast cancers (Fisher *et al.*, 1975). Between puberty and menopause the cyclic oestrogen and progesterone secretions ensure that susceptible cells are stimulated to divide, albeit in a cyclic manner. Hence, it is quite likely that during the course of protracted exposure to sparsely ionising radiation target cells will have had an opportunity to progress into the sensitive window.

Although the connection is not known between repair competence assayed by the $G_2$ lymphocyte test and those reparative processes that ordinarily reduce the induction of transformation and breast cancer, it is a possibility that the assay may underestimate the frequency of women who have an enhanced susceptibility. The reason is that repair related to target cell survival would also be involved. Enhanced cell killing, because of deficient repair, could give rise to increased compensatory repopulation and thus a greater flux of target cells into the sensitive window as sketched in Figure 3.

### Cancer incidence and protracted exposure to X- or γ-rays

The radiobiological model that has been described–kinetics in the absence of repair–is applicable to instances in which doses of high-LET radiation are protracted. Protraction of the exposure allows time for cells to progress into the sensitive window, late $G_2$/mitosis, during the normal maintenance of tissue function and integrity, or when induced to do so by compensatory mechanisms as well as by ancillary stimuli from other chemical or physical effectors. As for repair, in the instance of high-LET radiation a limited role is assured because of the qualitative differences in the intracellular damage from densely ionising particles compared with that from low-LET radiation.

As noted, for the model to have applicability to protracted exposure to low-LET radiation in addition to kinetics deficient repair is required. The latter property results from a characteristic of the cell and not the nature of the radiation damage. When repair deficiency obtains a concomitant requirement would be that the doses should be small. The reason is that cells in the sensitive window constitute the most sensitive moiety in the population and must survive the exposure to express transformation. Repair deficiency relative to transformation could be associated with a deficiency relative to survival. Hence, to observe enhanced tumorigenesis with a low-LET radiation a balance between cell killing and deficient repair would be required. In a given case the balance might require doses so small that the magnitude of the enhancement, resulting from kinetics during exposure, might not be detectable.

Increased cell division has been invoked as a cause of cancer because of the likelihood of molecular genetic errors and the subsequent altered growth control of premalignant and malignant cells (Preston-Martin *et al.*, 1990). The model described here is compatible with this possibility because even for protracted exposures to γ-rays, which can result in reductions in the yield of somatic mutations, altered genetic changes persist (Xing *et al.*, 1995).

**Acknowledgements**
This research was supported by the US Department of Health and Human Services, Public Health Service, via grant CA47497, and the US Nuclear Regulatory Commission via grant NRC-04-94-103.

### References

BALCER-KUBICZEK EK, HARRISON GH, TORRES BA AND MCCREADY WA. (1994). Application of the constant exposure time technique to transformation experiments with fission neutrons: failure to demonstrate dose-rate dependence. *Int. J. Radiat. Biol.*, **65**, 559–569.

BETTEGA D, CALZOLARI P, COSTA A, NORIS CHIORDA G AND TALLONE L. (1995). Oncogenic transformation of C3H 10T1/2 cells exposed to alpha particles: sensitivity through the cell cycle. *Radiat. Res.*, **142**, 276–280.

BOICE JD, PRESTON D, DAVIS FG AND MONSON RR. (1991). Frequent chest X-ray fluoroscopy and breast cancer incidence among tuberculosis patients in Massachusetts. *Radiat. Res.*, **125**, 214–222.

BORRESEN A-L, ANDERSEN TI, TRETLI S, HEIBERG A AND MOLLER P. (1990). Breast cancer and other cancers in Norwegian families with ataxia-telangiectasia. *Genes Chrom. Cancer*, **2**, 339–340.

BRENNER DJ AND HALL EJ. (1990). The inverse dose-rate effect for oncogenic transformation by neutrons and charged particles: a plausible interpretation consistent with published data. *Int. J. Radiat. Biol.*, **58**, 745–758.

CAO J, WELLS RL AND ELKIND MM. (1992). Enhanced sensitivity to neoplastic transformation of $G_2$-/M-phase cells exposed to $^{137}Cs$ γ-rays. *Int. J. Radiat. Biol.*, **62**, 191–199.

CAO J, WELLS RL AND ELKIND MM. (1993). Neoplastic transformation of C3H mouse embryo cells, 10T1/2: cell-cycle dependence for 50 kV X-rays and UV-B light. *Int. J. Radiat. Biol.*, **64**, 83–92.

CROSS FT. (1992). A review of experimental animal radon health effects data. In *Radiation Research*: A *Twentieth-Century Perspective*, Volume II: Congress Proceedings, Dewey WC, Edington M, Fry RJM, Hall EJ and Whitmore GF. (eds) pp. 476–481. Academic Press: San Diego, CA.

DARBY SC AND SAMET JN. (1994). Radon. In *Epidemiology of Lung Cancer*, Samet JM (ed.) pp. 219–243. Marcel Dekker: New York, NY.

DI MAJO V, COPPOLA M, REBESSI S, SARAN A, PAZZAGLIA S, PARISET L AND COVELLI V. (1994). Neutron-induced tumors in BC3F1 mice: effects of dose fractionation. *Radiat. Res.*, **138**, 252–259.

ELKIND MM. (1991). Physical, biophysical, and cell-biological factors that can contribute to enhanced neoplastic transformation by fission-spectrum neutrons. *Radiat. Res.*, **128**, S47–S52.

ELKIND MM. (1992). Enhanced neoplastic transformation by protracted exposures of high-LET radiations: a cell kinetic model based upon a $G_2$-/M-phase window of sensitivity. In *International Conference on Low Dose Irradiation and Biological Defense Mechanisms.* Aoyama T. (ed.) pp. 29–35. Elsevier: Amsterdam.

ELKIND MM. (1993). $G_2$/M-phase, a sensitive window for neoplastic transformation: implications of enhanced tumorigenesis by protracted low doses of fission-spectrum neutrons and other radiations. In *Fukui Workshop on Health Risks: Perspectives and Research.* Sugahara T, Torizuka K, Kobayashi S and Ishii Y. (eds), pp. 75–79. Health Research Foundation: Kyoto.

ELKIND MM. (1994). Radon-induced cancer: a cell-based model of tumorigenesis due to protracted exposures. *Int. J. Radiat. Biol.*, **66**, 649–653.

ELKIND MM AND HILL CK. (1986). Age-dependent variations in cellular susceptibility to neoplastic transformation: reply to letter to the editor by HH Rossi and AM Kellerer. *Int. J. Radiat. Biol.*, **50**, 1117–1122.

ELKIND MM AND SUTTON HA. (1959). X-ray damage and recovery in mammalian cells in culture. *Nature*, **184**, 1293–1295.

FISHER E, GREGORIO RM AND FISHER B. (1975). The pathology of invasive breast cancer: A syllabus derived from the findings of the National Surgical Adjuvant Breast Project. *Cancer*, **36**, 1–85.

GRAHN D, LOMBARD LS AND CARNES BA. (1992). The comparative tumorigenic effects of fission neutrons and cobalt-60 gamma rays in the BCF1 mouse. *Radiat. Res.*, **129**, 19–36.

HAN A AND ELKIND MM. (1979). Transformation of mouse C3H/$10T^1/_2$ cells by single and fractionated doses of X-rays and fission-spectrum neutrons. *Cancer Res.*, **39**, 123–130.

HAN A, HILL CK AND ELKIND MM. (1980). Repair of cell killing and neoplastic transformation at reduced dose rates of $^{60}Co$ γ-rays. *Cancer Res.*, **40**, 3328–3332.

HIEBER L, PONSEL G, ROOS H, FENNS, FRMKE E AND KELLERER AM. (1987). Absence of a dose-rate effect in the transformation of C3H 10T1/2 cells by alpha-rays. *Int. J. Radiat. Biol.*, **52**, 859–869.

HILL CK, BUONAGURO FM, MYERS CP, HAN A AND ELKIND MM. (1982). Fission-spectrum neutrons at reduced dose rates enhance neoplastic transformation. *Nature*, **298**, 67–69.

HILL CK, HAN A, BUONOGURO F AND ELKIND MM. (1984*a*). Multifractionation of $^{60}Co$ gamma-rays reduces neoplastic transformation in vitro. *Carcinogenesis*, **5**, 193–197.

HILL CK, HAN A AND ELKIND MM. (1984*b*). Fission-spectrum neutrons at a low dose rate enhance neoplastic transformation in the linear, low-dose region (0–10 cGy). *Int. J. Radiat. Biol.*, **46**, 11–16.

HILL CK, CARNES BA, HAN A AND ELKIND MM. (1985). Neoplastic transformation is enhanced by multiple low doses of fission-spectrum neutrons. *Radiat. Res.*, **102**, 404–410.

HILL CK, HAN A AND ELKIND MM. (1987). Promotion, dose rate, and repair processes in radiation-induced neoplastic transformation. *Radiat. Res.*, **109**, 347–351.

HOWE GR. (1995). Lung cancer mortality between 1950 and 1987 after exposure to fractionated moderate-dose-rate ionizing radiation in the Canadian fluoroscopy cohort study and a comparison with lung cancer mortality in the atomic bomb survivors. *Radiat. Res.*, **142**, 295 –304.

HOFFMAN DA, LONSTEIN JE, MORIN MM, VISSCHER W, HARRIS III, BSH AND BOICE Jr, JD. (1989). Breast cancer in women with scoliosis exposed to multiple diagnostic x rays. *J. Natl. Cancer Inst.*, **81**, 1307–1312.

HRUBEC Z, BOICE Jr JD, MONSON RR AND ROSENSTEIN M. (1989). Breast cancer after multiple chest fluoroscopies: second follow-up of Massachusetts women with tuberculosis. *Cancer Res.*, **49**, 229–234.

JONES CA, SEDITA BA, HILL CK AND ELKIND MM. (1989). Influence of dose rate on the transformation of Syrian hamster embryo cells by fission-spectrum neutrons. In *Low Dose Radiation*, Baverstock KF and Stather JW (eds) pp. 539–546. Taylor & Francis: London.

KAPLAN HS AND BROWN MB. (1952). A quantitative dose-response study of lymphoid-tumor development in irradiated C57 black mice. *J. Natl. Cancer Inst.*, **13**, 185–208.

LUBIN JH, BOICE Jr, JD, HORNUNG RW, EDLING C, HOWE GR, KUNZ E, KUSIAK RA, MORRISON HI, RADFORD EP, SAMET JM, TIRMARCHE M, WOODWARD A, XIANG YS AND PIERCE DA. (1994). *Radon and Lung Cancer Risk: A Joint Analysis of 11 Underground Miners Studies*. NIH Publication No. 94-3644. U.S. Department of Health and Human Services, National Institutes of Health, National Cancer Institution: Bethesda, MD.

MILLER RC, BRENNER DJ, GEARD CR, KOMATSU K, MARINO SA AND HALL EJ. (1988). Oncogenic transformation by fractionated doses of neutrons. *Radiat. Res.*, **114**, 589–598.

MILLER RC, RANDERS-PEHRSON G, HIEBER L, MARINO SA, RICHARDS M AND HALL EJ. (1993). The inverse dose-rate effect for oncogenic transformation by charged particles is dependent on linear energy density. *Radiat. Res.*, **133**, 360–364.

MILLER RC, GEARD CR, MARTIN SG, MARINO SA AND HALL EJ. (1995). Neutron-induced cell cycle-dependent oncogenic transformation of C3H 10T1/2 cells. *Radiat. Res.*, **142**, 270–275.

NAGASAWA H, KRAEMER KH, SHILOH Y AND LITTLE JB. (1987). Detection of ataxia telangiectasia heterozygous cell lines by postirradiation cumulative labeling index: Measurements with coded samples. *Cancer Res.*, **47**, 398–401.

PARSHAD R, SANFORD KK, JONES GM AND TARONE RE. (1985). $G_2$ chromosomal radiosensitivity of ataxia-telangiectasia heterozygotes. *Cancer Genet. Cytogenet.*, **14**, 163–168.

PIPPARD EC, HALL AJ, BARKER DJP AND BRIDGES BA. (1988). Cancer in homozygotes and heterozygotes of ataxia-telangiectasia and xeroderma pigmentosum in Britain. *Cancer Res.*, **48**, 2929–2932.

PRESTON-MARTIN S, PIKE MC, ROSS RK, JONES PA AND HENDERSON BE. (1990). Increased cell division as a cause of human cancer. *Cancer Res.*, **50**, 7415–7421.

REDPATH JL, HILL CK, JONES CA AND SUN C. (1990). Fission-neutron-induced expression of a tumour-associated antigen in human cell hybrids (HeLa × skin fibroblast): Evidence for increased expression at low dose rate. *Int. J. Radiat. Biol.*, **58**, 673–680.

ROSSI HH AND KELLERER AM. (1986). The dose rate dependence of oncogenic transformation by neutrons may be due to variation of response during the cell cycle. *Int. J. Radiat. Biol.*, **50**, 353–361.

SANFORD KK, PARSHAD R, PRICE FM, JONES GM, TARONE RE, EIERMAN L, HALE P AND WALDMAN TA. (1990). Enhanced chromatid damage in blood lymphocytes after $G_2$-phase X-irradiation, a marker of the ataxia-telangiectasia gene. *J. Natl. Cancer Inst.*, **82**, 1050–1054.

SARAN A, PAZZAGLIA S, COPPOLA M, REBESSI S, DI MAJO V, GARAVINI M AND COVELLI V. (1991). Absence of a dose-fractionation effect on neoplastic transformation induced by fission-spectrum neutrons in C3H 10T1/2 cells. *Radiat. Res.*, **126**, 343–348.

SARAN A, PAZZAGLIA S, PARISET L, REBESSI S, BROERSE JJ, ZOETELIEF J, DI MAJO V, COPPOLA M AND COVELLI V. (1994). Neoplastic transformation of C3H 10T1/2 cells: A study with fractionated doses of monoenergetic neutrons. *Radiat. Res.*, **138**, 246–251.

SCOTT D, SPREADBOROUGH A, LEVINE E AND ROBERTS SA. (1994). Genetic predisposition in breast cancer. *Lancet*, **344**, 1444.

SHILOH Y, PARSHAD R, FRYDMAN M, SANFORD KK, PORTNOI S, ZIV Y AND JONES GM. (1989). $G_2$ chromosomal radiosensitivity in families with ataxia-telangiectasia. *Hum. Genet.*, **84**, 15–18.

SHORE RE, HILDRETH N, WOODWARD E, DVORETSKY P, HEMPELMANN L AND PASTERNACK. (1986). Breast cancer among women given X-ray therapy for acute postpartum mastitis. *J. Natl Cancer Inst.*, **77**, 689–696.

SWIFT M, MORRELL D, MASSEY RB AND CHASE CL. (1991). Incidence of cancer in 161 families affected by ataxia-telangiectasia. *N. Engl. J. Med.*, **325**, 1831–1836.

TILL JE AND MCCULLOCH EA. (1963). Early repair processes in marrow cells irradiated and proliferating in vivo. *Radiat. Res.*, **18**, 96–105.

TILL JE AND MCCULLOCH EA. (1964). Repair processes in irradiated mouse hematopoietic tissue. *Ann. NY Acad. Sci.*, **114**, 115–125.

TOKUNAGA M, LAND CE, YAMAMOTO T, ASANO M, TOKUOKA S, EZAKI H AND NISHIMORI I. (1987). Incidence of female breast cancer among atomic bomb survivors, Hiroshima and Nagasaki, 1950–1980. *Radiat. Res.*, **112**, 243–272.

UPTON AC, RANDOLPH ML, CONKLIN JW, MELVILLE GS, CONTE FP, SPROUL JA, KASTENBAUM MA AND SLATER M. (1970). Late effects of fast neutrons and gamma rays in mice as influenced by the dose rate of irradiation: induction of neoplasia. *Radiat. Res.*, **41**, 467–491.

WELLS RL, CAO J, XING Y, HE L AND ELKIND MM. (1993). Transformation-sensitive cells in $G_2$-/M-phase are not promoted by TPA following $^{137}$Cs γ-rays. *Int. J. Radiat. Biol.*, **64**, 727–730.

XING Y, LINDQUIST K, LIU J, CROMPTON NEA, KITANI H, PATEL TC, MARTIN SG AND ELKIND MM. (1995). Low-dose-rate dependence of the phenotypic and genotypic expressions of mutagenesis by $^{137}$Cs γ-rays. *Radiation Oncology Investigations*, **3**, 17–28.